# Understanding experiences of mental health help-seeking in Arab populations around the world: a systematic review and narrative synthesis

**DOI:** 10.1186/s12888-023-04827-4

**Published:** 2023-05-09

**Authors:** Hania El Khatib, Aisha Alyafei, Madiha Shaikh

**Affiliations:** 1 Division of Psychiatry, University College London, London, UK; 2 Research Department of Clinical, Educational and Health Psychology, University College London, London, UK; 3 North East London NHS Foundation Trust, London, UK

**Keywords:** Help-seeking, Arabs, Behaviours, Attitudes, Barriers, Facilitators

## Abstract

**Background:**

Racial and ethnic disparities in mental health service utilisation and access is well established. Mental illness is common among Arab populations globally, but most individuals display negative attitudes towards mental health and do not seek professional help. The aim of this systematic review was to determine 1) help-seeking behaviours 2) help-seeking attitudes and 3) help-seeking barriers and facilitators, related to mental health services among Arab adults.

**Method:**

A pre-defined search strategy and eligibility criteria allowed for database searching using terms related to: mental health, Arabs, help-seeking, as well as experiences and behaviours. Seventy-four articles were included and analysed through narrative synthesis. Results were reported using the PRISMA guidelines. The review protocol was registered prospectively on PROSPERO (CRD42022319889).

**Results:**

Arabs across the world have negative attitudes towards formal help-seeking and are reluctant to seek help, despite the presence of psychological distress. There is little information on factors that influence help-seeking behaviours and rates of service use. Preference for informal help sources such as family and friends were expressed and considered more acceptable. Low mental health literacy, stigma, gender, age, education, religion, acculturation, and immigrant status were the most common factors influencing help-seeking attitudes. Barriers to help-seeking included stigma, privacy and confidentiality, trust, mental health literacy, language, logistics, and culture related barriers. Increasing societal and family awareness, external support and encouragement, shared culture between the client and therapist, quality of doctor patient relationship, and feelings of connectedness with the host country among refugees were mentioned facilitators. Mixed findings for the role of religion, and family and community, in relation to facilitating or hindering help-seeking were reported.

**Conclusions:**

There is an increased likelihood and preference to seek informal sources of psychological support among Arabs. Contextual and cultural factors impeding help-seeking for Arabs are common across the world. Future research should address actual utilisation rates of services to better understand factors that influence help-seeking behaviours and facilitators to help-seeking. Increasing mental health literacy and developing anti stigma campaigns is necessary. Developing culturally informed interventions should inform future efforts to promote help-seeking among this population.

**Supplementary Information:**

The online version contains supplementary material available at 10.1186/s12888-023-04827-4.

## Background

There is an increasing acknowledgement of the importance of mental health as a leading cause of disability which has significant effects on daily functioning [1]. There is a need for mental health support, however, the gap between the number of individuals needing care and those seeking help or receiving services remains substantial [2]. Of the people who need support or treatment, only an estimated 10% receive the necessary help [3]. Rickwood and Thomas [4], propose that there is no agreed and commonly used definition or measurement for help-seeking. Help-seeking is generally understood as an adaptive coping process of seeking external assistance to deal with mental health difficulties, including formal (e.g., psychologists and psychiatrists) and informal (friends and family) help [5]. According to Rothi and Leavey [6], help-seeking progresses through three phases: a) recognition, b) decision and c) action. Additionally, Andersen’s healthcare utilisation model draws on three dynamics determining the usage of health services: *Predisposing factors* such as race. age, and health beliefs, *enabling factors* such as family support, and *need,* including actual or perceived need for care [7]. While these models appear to operate in simple stages on the surface, numerous factors facilitating or obstructing this process are complex and poorly understood. Moreover, identifying and responding to mental health difficulties largely varies, and mental health help-seeking experiences differ across contexts and populations.

There is disparity in the use of and access to mental health services in racial and ethnic minority populations partly due to stigma and psychocultural variables in seeking psychological help [8–10]. A recent systematic review established that Filipinos across the world have general reluctance and unfavourable attitude towards formal help-seeking despite high rates of psychological distress, due to cultural, logistical barriers and immigration experiences in host countries [11]. Language, lack of awareness of available services, stigma and negative attitudes towards treatment and providers, are highlighted barriers to access to mental health services for refugees and asylum seekers in Europe [12]. Similar findings in existing Arab mental health literature demonstrate that individuals tend to underutilize mental health services, displaying negative attitudes toward mental health and psychological services [13]. Although help-seeking among Arabs has not been substantially researched, Arab culture influences the definition and conceptualization of psychiatric disorders [14]. A systematic review identified the prevalence of stigmatising beliefs and attitudes toward treatment of mental illness in patients, care providers, and the public, highlighting reluctance to seek help among this population [15].

The Arab world includes diverse groups of Arabic speaking individuals who share similar cultural values, originating from 22 Arab countries including: Lebanon, Syria, Egypt, Palestine, Iraq, Algeria, Bahrain, the Comoros Islands, Djibouti, Jordan, Kuwait, Libya, Morocco, Mauritania, Oman, Qatar, Saudi Arabia, Somalia, Sudan, Tunisia, the United Arab Emirates (UAE), and Yemen [16, 17]. This group has a combined population of over 400 million, and the number of international Arab migrants increased from 19 to 34 million in 10 years [18]. Little is known about the mental health of Arabs, even with its large population size. There is a lack of updated reliable epidemiological psychiatric data and very little published on national lifetime prevalence and treatment of mental disorders in the region [19]. Studies have so far focused on small populations in Egypt, Morocco, Iraq, Morocco, Qatar, and UAE, establishing comparable rates of psychological disorders to western countries [20]. However, studies report substantial delays between the onset of disorders and onset of treatment, and highlight that only a minority of Arabs with any mental disorder ever received professional treatment [19].

To date, a systematic review found that in addition to stigma, factors that affect attitude toward counselling among Arabs include barriers such as fear of self-disclosing, traditional healing methods, mental illness conceptualization, culture, family, and religious leaders [21]. This review also documented that Arabs visit psychologists, counsellors, and psychiatrists, less than other ethnic groups. While interesting, this review only included papers from 2003 to 2017, and participants were limited to students and hospital patients. Moreover, the review did not mention all 22 Arab countries, making it challenging to compare Arabs residing in the Middle East, North Africa, and other Western countries.

Despite the evident psychological distress in Arab populations, treatment rates and help seeking remain low [22]. Studies considered attitudes and factors influencing help seeking among Arabs as well as service utilisation patterns, but experiences of mental health help seeking have not been reviewed systematically. The literature has not considered how help seeking experiences and deterrents to seeking help differ for Arab individuals in different geographical contexts. Importantly, most of the research focuses solely on barriers to help seeking rather than facilitators. There is limited knowledge on why help seeking remains low for Arabs across different countries and how to promote it remains unclear.

In turn, understanding Arab mental health help-seeking behaviours will aid to improve service uptake and reduce mental health burden. The number of international Arab migrants and refugees increased considerably in the last few years [23], as armed conflicts and political instability in countries of origin forced many to relocate, significantly impacting their mental health [24]. Post migration stressors leave individuals particularly vulnerable to mental health illnesses because of language difficulties, cultural isolation, and socioeconomic factors shaping their experiences in new settings [25, 26]. The health of international Arab migrants has become both a global and a regional public health issue, as cultural and religious differences between migrants and host country populations exacerbate this [23]. Capturing the heterogeneity of help seeking experiences among Arab diaspora can inform interventions promoting help seeking and improve quality of psychological support for Arabs in different regions of the world. Additionally, exploring help seeking behaviours and attitudes from the perspective of both professionals and individuals themselves, is critical to bridging the gap between evident mental health difficulties and formal mental health services.

### Aims & objectives

The aim of this review is to understand experiences of mental health help seeking in Arab adults across different geographical contexts. The paper focuses on Arabs in different regions, identifying: 1) help-seeking behaviours, 2) help-seeking attitudes, and 3) help-seeking barriers and facilitators, relating to mental health.

## Methods

This review was registered on PROSPERO (CRD42022319889), and reported in accordance with the PRISMA guidelines [27].

### Search strategy

An initial search was performed in March 2022 and then updated on June 1^st^, 2022 to include papers published from inception up to that date. Five databases were systematically searched including MEDLINE, PsycINFO, Embase, CINAHL, and ProQuest Middle East & African database. Grey literature from the mentioned databases was included. The search terms were developed using PICO framework and synonymous text words and MeSH terms were used to inform search terms associated with the following four concepts: 1) Mental Health, 2) Arabs, 3) Help Seeking, 4) Experiences and behaviours. The search was limited to English, and search terms were tailored to each database and combined using Boolean operators. Studies identified in this process were exported to EndNote 20 and then to Covidence (a systematic review manager). Duplicate papers were removed, and a priori inclusion/ exclusion criteria applied to initial screening of titles and abstracts, and then for full text review. Full text papers that could not be retrieved were accessed upon request from the UCL library services. 50% of the included full text papers were screened by an independent reviewer. Any conflicts were resolved in consultation with two independent reviewers (MS & AA).

### Eligibility criteria

#### Inclusion

Both qualitative and quantitative studies on mental health help-seeking behaviours, attitudes, and facilitators or barriers to help-seeking were included. Studies reporting on factors that influence mental health help-seeking in Arabs were also included. Mixed-methods studies were included when providing relevant qualitative and/or quantitative data. This review included papers that reported on these outcomes even if not identified a priori as a research objective. Help-seeking was understood as seeking help from informal sources (family, friends, online sources, self-help resources) or formal treatment (GP, mental health professionals, etc.). Participants had to be 18 years or older and from Arab ethnicities from any of the 22: (Bahrain, Lebanon, Jordan, Iraq, Oman, Qatar, Saudi Arabia, Syria, Egypt, Palestine, Kuwait, UAE, Yemen, Algeria, Libya, Morocco, Somalia, Sudan, Tunisia, Djibouti, Comoros, Mauritania) and were considered regardless of geographical location, gender, or religion. Papers with immigrant and refugee Arab populations were included to capture similarities and differences in experiences compared to those residing in Arab countries. The review included the perspectives of clinicians and carers to understand their different perspectives, if any. All studies on Arabs and mental health help seeking were included ranging from inpatients, outpatients, and other informal sources to those that have never sought support or services. For this review, no limitations were placed on the type of mental illness or concern to capture experiences and perspectives of non-diagnosed individuals as well.

#### Exclusion

Papers that addressed help-seeking in relation to non-mental health concerns and/organic causes such as physical or developmental illnesses were excluded. Studies with participants below 18 years of age or with parents seeking help for their children were excluded. Studies were excluded if data for Arab participants could not be extracted independently from the rest of the sample. Articles written in languages other than English, literature and commentary reviews, protocols, and case studies were excluded.

#### Data extraction

Data for included studies were extracted using a predefined form and identified: Author, year of publication, country, study design, sample number, demographic characteristics (age/nationality), outcomes, and key findings on attitudes, behaviours, and factors influencing mental health help seeking.

#### Quality assessment

The Mixed Methods Appraisal Tool (MMAT) [28] previously validated and used in various mixed methods systematic reviews [29] was used for quality assessment, given the variety of study designs included in this review. The MMAT included a specific checklist to evaluate main features of each study design. Each checklist had criteria to evaluate the most relevant aspects of the specific study design (e.g.: for qualitative papers, “was the qualitative data collection method adequate to address the research question?”) with possible responses of “yes”, “no”, and “can’t tell”. An independent second reviewer (AB) rated 10% of included studies for quality assessment, assessing an equal number of papers from each study design. Overall study quality was not used as an exclusion criterion because we opted to be overly inclusive to gain a sense of the methodological quality of available evidence and provide a comprehensive overview of mental health help-seeking experiences in Arabs across countries.

#### Data synthesis

A narrative synthesis was adopted to synthesise results by describing, comparing, and combining findings across included studies [30]. Due to the overlap between quantitative and qualitative data, findings were identified and grouped based on the outcomes of the review: help-seeking behaviours, attitudes, and barriers and facilitators. Results for each outcome were discussed by organising findings into prominent themes evident across studies. Differences and similarities of help seeking experiences for Arabs immigrants and refugees compared to Arabs in Arab countries were highlighted. Relevant findings are discussed from various perspectives such as mental health professionals and carers when applicable.

## Results

**Fig. 1:**
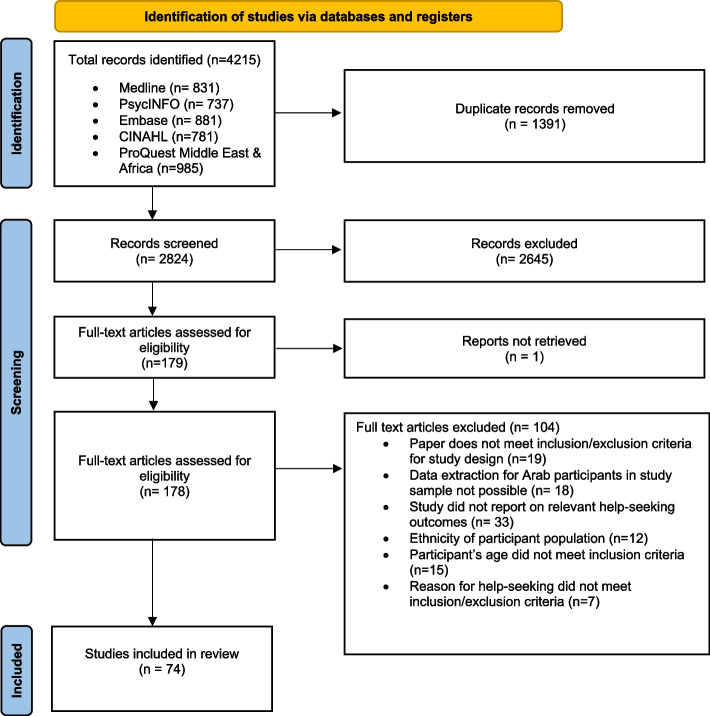
PRISMA diagram. Flowchart for study selection and inclusion

A total of 4215 papers were identified through database searching. After removing duplicates, 2824 papers were screened for titles and abstracts, and 2645 records were excluded. Access to one paper was not provided during the screening period and 179 articles were then retrieved for full-text screening. A total of 74 studies were eligible for inclusion. Reasons for exclusion of full-text papers after the screening stage and details of the literature search are reported in Fig. 1.

### Characteristics of included studies

The final 74 studies in this review included qualitative (*n* = 28), quantitative (*n* = 41), and five mixed methods studies. Nine PhD dissertations were retrieved from the initial search and included as grey literature. The characteristics of each study are presented in Table 1. Most of the studies were conducted in the United States (*n* = 12), followed by Israel (*n* = 8), and both Jordan (*n* = 6) and Australia (*n* = 6). One study collected data in three countries (Palestine, Egypt, and Kuwait), and one study was conducted online across several countries. All studies were published between 2002 and 2022.

**Table 1:** Characteristics of included studies

Author(s) (Year)	Study Country	Study Design	Sample Size (N)	Age and Participants	Aim/Outcome(s)	Key Findings	
Aarethun et al. (2021) [31]	Norway	Qualitative	31	> 18 year old Syrian Refugees	Help-seeking preferences for PTSD and depression	Social network preferred help-seeking source Cultural stigma barrier to formal help Barriers to help-seeking in Norway: miscommunication, language, trust Help-seeking affected by migration process and is contextual	
Abubotain (2020) [32]	USA	Quantitative	102	> 18 year old first and second generation Arab Americans	Factors involved in attitudes toward seeking professional mental health services Differences between the way first and second generation Arab Americans seek and perceive professional help	Acculturation, stigma, gender involved in help seeking Second generation more likely to utilize mental health services First generation more likely to use culturally accepted means (Friends, family) Societal stigma towards seeking mental health support in both groups	
Abuhammad & Hamaideh (2022) [33]	Jordan	Quantitative	205	> 20 year old Jordanian nursing students	Attitudes toward seeking professional psychological help, before and after taking part in a mental health course	Positive effect of mental health course on attitudes toward seeking professional psychological help	
Ahmed et al. (2017) [34]	Canada	Mixed Methods	12	> 20 year old Syrian refugee pregnant or postpartum women	Barriers to access maternal mental health services and seek help	Stigma, privacy concerns, language	
Alajlan (2016) [35]	USA	Mixed Methods	197	> 19 year old Saudi Arabian international students	Relationship between psychological attitudes toward mental health services and gender, age, education, marital status, counselor type Barriers to seeking counseling or psychotherapy	Counseling or psychotherapy experience described as positive Enthusiasm about seeking mental health services Barriers: counselor’s cultural insensitivity, discrimination and fear of being incriminated for being from the Middle East	
Al Ali et al. (2017) [36]	Jordan	Quantitative	428	> 18 year old Jordanians attending primary health care centers	Factors influencing attitudes and preferences toward seeking formal mental health services	Tendency to seek informal mental health resources and less favorable attitude to seeking formal help Stigma, cultural beliefs about mental health problem, institutional barriers	
Al-Busaidi (2010) [37]	Oman	Qualitative	30	> 21 year old Omani women attending primary health care > 31 year old Omani GPs	Attitudes and beliefs regarding help seeking behavior for emotional distress	Informal source of help preferred (faith, family, traditional healer) Quality of doctor patient relationship influences likelihood of seeking/continuing treatment Stigma, time restraints	
Al-Darmaki et al. (2016) [38]	UAE	Qualitative	70	> 18 year old Emirati female college students	Help seeking attitudes/barriers	Willingness among majority to seek help for psychological problems Barriers: social stigma, misconception of role of psychologists or services, lack of awareness or knowledge of psychological disorders, mistrust, and lack of confidence in professionals, financial difficulties	
Al-Dousari & Prior (2020) [39]	Kuwait	Qualitative	3	> 28 year old Kuwaiti women receiving counselling	Perspectives on decisions to seek help/experiences of help-seeking	Faith facilitated and supported formal help-seeking	
Alhomaizi et al. (2018) [40]	USA	Qualitative	17	Lay informants: M = 26 years, Key informants: M = 42, Arab Muslims	Factors influencing decision to seek formal MH treatment	Stigma Family/community role and support Mental health literacy, beliefs about causes of mental health problems, gender	
Ali & Agyapong (2016) [41]	Sudan	Mixed Methods	109	> 20 year old carers of mentally ill patients and psychiatrists	Barriers to mental health services utilization	Barriers: beliefs around mental illness, resorting to alternative treatments (religious and traditional healers), centralization of mental health services, inadequate number of mental health staff, and mental health not being a priority by policy makers Psychiatric consultants identified stigma, cost of medications, and worries about medication’s side effects	
Alissa (2021) [42]	Saudi Arabia	Quantitative	1632	> 18 year old Saudi Arabians (3% non-Saudi)	Impact of social barriers on mental health help-seeking	Majority of the participants agreed that social barriers could prevent them from seeking mental health help Stigma chosen as the most common social barrier, followed by culture and negative perceptions of mental illness	
Alkhayat-Hatahet (2021) [43]	USA	Quantitative	63	> 18 year old Syrian Refugees in Southeast Michigan	Barriers to receiving or seeking MH services Association between perceived needs for mental health services and utilized services	Barriers: lack of information about services available, judgement of the community and shame, language, lack of previous experience in these services Perceived need for services but low utilization	
Al-Krenawi (2002) [44]	Israel	Quantitative	15,698	Age not specified- National hospital records of Arabs	Mental health service utilization patterns	Arab women utilize psychiatric services less than Arab men Arabs underutilize mental health services	
Al-Krenawi et al. (2009) [45]	Egypt Kuwait Palestine	Quantitative	716	M = 22 year old Israeli-Arab, Egyptian, Kuwaiti, and Palestinian students	Perceptions and attitudes towards help-seeking	Higher intention to consult professional MH services among woman Differences in perceptions and attitudes between Palestinians and Arab-Israelis compared to Kuwaitis and Egyptians	
Al-Krenawi & Graham, (2011) [46]	Israel	Quantitative	195	> 18 year old Arab university students	Attitudes and mental health-seeking patterns in 3 major religious minorities	Higher interpersonal openness, less stigmata towards services, less likely to use traditional healing systems in Christians compared to Druze and Muslims	
Al-Krenawi et al. (2004) [47]	Israël	Quantitative	262	> 19 year old undergraduate female university students from Jordan, UAE, Israel	Attitudes to mental health treatment	Age, educational attainment, marital status predictors of attitudes to help-seeking Faith common help-seeking behavior in times of distress Nationally not a predictor to attitudes of MH treatment	
AlLaham et al. (2020) [48]	Lebanon	Qualitative	46	> 18 year old Syrian Refugees and Lebanese community members	Factors that influence help-seeking behaviors	Lack of knowledge about mental health symptoms and available services, stigma, financial barriers Less stigma to seeking help from religious leaders	
Aloud & Rathur (2009) [49]	USA	Quantitative	286	> 18 year old Arab Muslims in Ohio USA	Attitudes toward seeking and using formal mental health and psychological services	Attitudes towards seeking and formal MH services influenced by cultural and traditional beliefs about MH problems, knowledge and familiarity of services, perceived societal stigma, use of informal services	
Al-Roubaiy et al. (2017) [50]	Sweden	Qualitative	10	> 21 year old Male Iraqi refugees	Attitudes and experiences of counselling	Positive and negative views/experiences to counselling Social support valued Reluctance to disclose issues to therapists Barriers: transparency and competence of therapists	
Al-Soleiti et al. (2021) [51]	Jordan	Qualitative	20	> 24 year old Jordanian and Syrian Mental health professionals working with refugees	Barriers to seeking mental health treatment and ability of local systems to provide services	Barriers: Financial, stigma, systemic/organizational (distrust in the system, accessibility, misdiagnosis, and lack of screening in primary settings, shortage of mental health professionals, communication and language, legal/immigration barriers), stigma, awareness and education, limited accessibility	
Ayalon et al. (2015) [52]	Israel	Qualitative	45	> 20 year old Israeli Arab women, primary care patients, primary care providers	Attitudes/use of alternative services and informal help-seeking behaviors for depression and anxiety	3 informal help-seeking behaviors identified: social support (extended family and neighbors versus nuclear family and close friends) religiosity, self-help techniques Primary care providers did not endorse religion and self-help	
Balesh et al. (2018) [53]	USA	Quantitative	298	> 18 year old Arab Americans	Effects of acculturation, ethnic identity, and spirituality on mental health service utilization attitudes	Greater levels of identity and heritage and mainstream acculturation predictors of higher levels and willingness to seek help	
Bashir et al. (2020) [54]	Sudan	Quantitative	644	M = 20 year old Medical students	Mental health care seeking behaviors and barriers	Barriers: Fear of stigmatization, preference for dealing with the problem alone, fear of the unknown, and failure to recognize symptoms	
Bawadi et al. (2022) [55]	Jordan	Qualitative	24	> 18 year old Syrian refugees and community leaders	Barriers and facilitators to the use of mental health services	Barriers: Lack of awareness of mental illness and available services Availability, accessibility, and affordability of mental health services Stigma and social discrimination	
Castaneda et al. (2020) [56]	Finland	Quantitative	512	> 18 year old Somali migrants in Finland	Mental health-related use of health services and the correspondence between the need and use of services	High need for services but low use of mental health services Lack of awareness/familiarity with services	
Dogan et al. (2019) [57]	Turkey	Qualitative	24	> 18 year old Syrian Refugees	Experiences and difficulties regarding mental health services	Difficulties making appointments, obtaining medicine, personal rights, lack of information, language, discrimination,	
Elghoroury (2017) [58]	USA	Quantitative	131	> 18 year old Muslim Arab Americans	Relationship of acculturation and religiosity on help-seeking attitudes	No relationship between acculturation and religiosity on help-seeking attitudes Gender and age predictors of help-seeking attitudes	
Fassaert et al. (2009) [59]	Netherlands	Quantitative	127	> 18 year old Moroccan migrants in Netherlands	Uptake of mental health services	Moroccan migrants did not differ in uptake of specialized mental health services compared to other non-Arab ethnicities in presence of a common mental disorder Difference in uptake for psychological distress. Moroccan migrants less likely to report uptake of primary care for mental health problems	
Fekih-Romdhane et al.(2021) [60]	Tunis	Quantitative	714	> 18 year old Tunisian students	Association between stigma levels and help-seeking intentions and comfort with disclosing mental illness	Better knowledge of mental illness predicted favorable help-seeking intentions Favorable help seeking intentions associated with lower stigma Gender predictor of help seeking intentions: females higher in help-seeking intentions and knowledge of mental illness Comfort with disclosing significantly and negatively correlated with attitudes to help seeking	
Fuhr et al. (2020) [61]	Turkey	Quantitative	1678	> 18 year old Syrian Refugees	Mental health care utilisation and barriers to seeking and continuing care	Structural and attitudinal barriers for not seeking care: cost of mental health care, the belief that time would improve symptoms, fear of being stigmatized and lack of knowledge on where and how to get help	
Gundel et al. (2016) [62]	USA	Qualitative	9	> 27 year old Sudanese refugees	Factors promoting seeking mental health care and beliefs about counselling	Religion, education, age Use of help from community members Distrust and Ambivalence for Western Mental Health Care Barriers to Western Mental Health Care: confidentiality and stigmatization of mental health, experiences as refugees and as a cultural minority in dominant culture, assumed lack of multicultural competence ascribed to mental health practitioners	
Habhab (2018) [63]	USA	Qualitative	11	> 25 year old Arab American psychotherapists	Barriers and facilitators of providing mental health services	Barriers: acculturation, gender, family support, stigma and community	
Hamid & Furnham (2013) [64]	UK	Quantitative	259	> 18 year old Arabs living in UK	Factors affecting attitude towards seeking professional psychological help	Less positive attitudes towards seeking professional psychological help Education, age, years in host country and confidentiality concerns predictors of attitudes to help seeking Shame and Gender not predictors	
Harris et al. (2021) [65]	Norway	Quantitative	92	> 18 year old Syrian refugees	Help-seeking preferences and perceived barriers in accessing help from the GP	Preference to seek help from informal sources mostly family, partner, and God, followed by seeking help from GP Barriers: language, perceiving services as unhelpful, long wait times, not feeling understood Feelings of connectedness with host country and social integration promote help-seeking from GP	
Hasan & Musleh (2017) [66]	Jordan	Qualitative	27	> 37 year old family members of patients with psychosis	Barriers to seeking early psychiatric treatment	Barriers: perceived stigma, role of extended family members, financial reasons, misattribution of the cause and symptoms of mental illness	
Kamel et al. (2021) [67]	Egypt	Quantitative	707	M = 20 year old Egyptian medical students	Help-seeking behaviours and barriers to accessing care	Preferred sources of help in order: self-help, family/friends, professional services last Barriers: preferred to handle problem alone, did not know where to go, stigma/infrastructural barriers (wait times, travel distance, not having needs met)	
Karadag et al. (2021) [68]	Turkey	Mixed-Methods	440	Age not specified- Syrian refugees in Turkey	Challenges and experiences in assessing mental health problems and barriers to accessing mental health care	Barriers: language, lack of knowledge about existing services Health provider's mentioned barriers: higher prioritization of daily life challenges, physical health problems and their low level of awareness on available services	
Karam et al. (2018) [69]	Lebanon	Quantitative	2857	> 18 year old Arab adults	Determinants and barriers of seeking help for mental disorders	Barriers: perceived severity of problem and perceived need, financial, uncertainty about where to go and who to see, logistic (transportation/appointments), dissatisfied with previous treatment Female gender, higher education, and income predictors of positive attitudes to help seeking	
Kayrouz et al. (2018) [70]	Online	Quantitative	503	> 18 year old Arabs	Acceptability of traditional face-to-face and internet delivered mental health services Barriers to services	Moderate to high acceptability rates for mental health services Differences in acceptability of mental health services by country Females more likely to try internet delivered treatment compared to males	
Kayrouz et al. (2015) [71]	Australia	Quantitative	252	> 18 year old Arab Australians	Help-seeking behaviors and barriers to accessing psychological treatments		Barriers: shame, trust, mental health literacy, practical barriers (time, cost, transport) No differences by country of birth, gender, or religion Preference for non-medical support Low rates of service use
Khatib & Abo-Rass (2021) [72]	Israel	Qualitative	28	> 18 year old Arab students	Mental health literacy		High levels of mental health literacy among students and knowledge of available services Preference for self-treatment and religious therapies Pessimistic attitudes to help seeking Barriers: language and stigma
Kiselev et al. (2020) [73]	Switzerland	Qualitative	5	> 18 year old Syrian Refugees	Structural and socio-cultural barriers to accessing mental healthcare		Basic needs prioritized, gender, mismatch between western system of diagnosis and needs perceived by refugees, stigma, lack of awareness of services, lack of resources, language, lack of awareness of the health system
Levav et al. (2007) [74]	Israel	Quantitative	632	> 21 year old Arab-Israelis	Rate of help-seeking		Lower rates of help-seeking compared to Jewish Israeli’s and no perception of need for it
Linney et al. (2020) [75]	UK	Qualitative	23	> 18 year old Somali's living in UK	Views on accessing appropriate healthcare and ideas to improve access and reduce barriers		Religious healing, medication, community support and services expressed as potentially helpful treatments Barriers: unsure where to access services, language barriers, long waiting times and a lack of continuity with seeing different medical professionals, mistrust of perceived authority figures and fear of going to the doctor or getting treated due to potential economic repercussions
Loewenthal et al. (2012) [76]	UK	Qualitative	24	> 40 year old Somali's living in the UK	Understanding of mental health issues and available services Barriers to accessing of psychological therapies services		Lack of understanding of Western conceptualizations of depression/anxiety Lack of appropriate knowledge of services and fear of repercussions if accessed services Barriers: confidentiality (interpreters), stigma, needs not addressed (language and cultural barriers) Religion as method to deal with mental health issue
Mahajan et al. (2022) [77]	Canada	Qualitative	12	> 19 year old Syrian refugee women	Roles of social networks in resource seeking behaviors		Family role in providing information about Canada’s health system Social networks influence assumptions about mental health services Women feel more welcomed into social networks in Canada than in countries of first asylum. Social networks as alternatives to seeking formal help
Mahmoud (2018) [78]	Saudi Arabia	Quantitative	5644	> 20 year old Saudi Arabian adults	Knowledge, attitudes and perceptions towards health services and barriers that affect willingness to seek psychiatric help		Many unaware about psychiatric services available in the kingdom Reports of not seeking help when needed psychiatric help when needed Shame felt in relation to help-seeking Male gender, > 20 years old, not knowing whether a relative is suffering from mental illness and not knowing about the services provided by psychiatric health services associated with unwillingness to seek psychiatrist consultation Gaps in knowledge of mental illness
Mahsoon et al. (2020) [79]	Saudi Arabia	Quantitative	236	> 18 year old Saudi Arabians	Attitudes to MH help seeking Relationships between parental support, beliefs toward mental illness, and mental help-seeking attitude		Highly positive attitude toward mental help-seeking No relationship between parental support, beliefs towards mental illness and mental-help seeking
Mamdouh et al. (2022) [80]	Egypt	Quantitative	707	M = 20 year old Egyptian students	Attitudes, interest, and perceived barriers to electronic mental services		Little knowledge about electronic MH Barriers: privacy and confidentiality, unfamiliarity, technical issues
Markova & Sandal (2016) [81]	Norway	Mixed Methods	105	> 18 year old Somali refugees	Understand preferred coping strategies (in this paper refers to the way in which people prefer to react to or deal with depression, including help seeking behavior and preferred treatment)		Strong preference for coping with depression by religious practices and reliance on family, friends, and religious community, rather than by seeking professional treatment from public health services
Markova et al. (2020) [82]	Norway	Quantitative	100	M = 30 year old Somali immigrants in Norway	Preferred help-seeking sources for depression		Endorsement of traditional help sources and informal help Acculturation and education influenced help-seeking preferences
McKell et al. (2017) [83]	Jordan	Qualitative	16	Age not specified- Palestinian refugees	Barriers to accessing and consuming mental health services		Barriers: resource and financial deficits, sex, stigma, religion, culture, and discrimination
Molsa et al. (2010) [84]	Finland	Qualitative	27	> 50 year old Somali migrants in Finland	Change in help-seeking practices and use of services		Importance of trust in help-seeking Religious figures primary source of help Cultural barriers to treatment include not feeling understood by healthcare professionals
Molsa et al. (2019) [85]	Finland	Quantitative	128	> 50 year old Somali migrants	Healthcare services utilization patterns and preferences for mental healthcare		Low use and access to services Preference of traditional care and religious healing High level of symptoms not associated with use of services
Mond et al. (2021) [86]	Australia	Quantitative	66	> 18 year old Iraqi refugees	Association between trauma-related psychopathology recognition and help-seeking		Self-recognition of symptomology associated with help seeking Poor self-recognition level of trauma-related psychopathology barrier to help seeking
Nazzal (2015) [87]	USA	Quantitative	166	> 18 year old Arab Americans	Impact of biculturalism on well- being, race-related stress, and perceptions of a racist environment on attitudes towards help-seeking		No significant effect of biculturalism, well- being, race-related stress, and perceptions of a racist environment on attitudes towards seeking professional psychological help
Noorwali et al. (2022) [88]	Saudi Arabia	Qualitative	12	> 21 year old Saudi Arabians	Barriers and facilitators of seeking mental health support		Barriers: public stigma and lack of awareness, unprofessional practitioners, lack of accessibility to services and information, unsupportive families, intrapersonal dilemmas, and misconceptions based on religious beliefs Facilitators: increasing societal and family awareness, promoting the accessibility of services, enhancing sources of external support, personal motivation to change, and online therapy
Noubani et al. (2020) [89]	Lebanon	Qualitative	36	> 18 year old Lebanese hosts and Syrian refugee community members	Health seeking behaviors and barriers to health access		Women more likely to seek support Informal help sources first choice of support Barriers: significant delays in seeking help from formal services, social stigma, service costs, lack of health coverage, limited awareness of service availability, limited trust in the quality of services available
Palgi et al. (2011) [90]	Israel	Quantitative	1068	> 20 year old Arabs in Israel after war with Lebanon	Association between demographic variables, war-related factors, and psychosocial factors and mental health utilization		Injury of a relative during the war associated with increased probability of mental health service utilization No effect of gender, marital status, distress symptoms on service use
Piwowarczyk et al. (2014) [91]	USA	Mixed Methods	16	> 18 year old Somali refugees or citizens in the US	Attitudes and beliefs about treatment Barriers to accessing mental health services		Turning to family or friends for support rather than acquiring formal services Traditional ways of healing and coping/ religion Negative attitudes toward medication Barriers: Western mental health services and mental health needs from a western perspective not understood, disclosure to strangers, stigma
Rae (2014) [92]	UK	Qualitative	12	> 20 year old Somali male refugees in UK	Views on Western-based professional help and barriers to treatment		Barriers: stigma, difficulty disclosing (fear of judgement, privacy, cultural norms of enduring difficulties), GP lack awareness of cultural backgrounds, fear of diagnosis and treatment, Traditional healing preferred method of treatment
Rakhawy (2010) [93]	Egypt	Quantitative	5191	> 18 year old Egyptian adults	Frequency and use of faith healing		Help-seeking tendency directed towards primary health care services first, followed by relatives, faith healers
Said et al. (2021) [94]	Australia	Qualitative	31	> 18 year old Somali-Australian women	Perceived barriers to help-seeking for mental health		Barriers: Influence of faith, stigma, mistrust of Western healthcare system and denial of mental illness
Schlechter et al. (2021) [95]	Germany	Quantitative	384	> 18 year old Syrian refugees	Attitudes toward seeking professional psychological help		More negative attitudes toward professional psychological help-seeking
Schubert et al. (2019) [96]	Finland	Quantitative	351	18–64 year old Somali immigrants in Finland	Association of psychosocial factors (traumatic events, social network, acculturation indices, mental health, and trust in services) with help-seeking		Past traumatic events increase use of MH services Gender (men) utilized mental health services more
Shechtman et al. (2018) [97]	Israel	Quantitative	196	> 18 Clinical and non-clinical Arab adults	Mediation of help-seeking stigma towards group therapy		Higher public stigma linked to higher self-stigma, and in turn decreased intention to seek group therapy
Slewa-Younan et al. (2015) [98]	Australia	Quantitative	225	> 18 year old Iraqi refugees	Levels of psychological distress and help seeking behavior Associations between mental health and help seeking and demographic characteristics		High levels of distress yet low uptake of mental health treatment Association between help seeking behavior and PTSD symptomology No association between age, sex, religion, education, marital status, distress levels
Smith (2011) [99]	USA	Qualitative	14	> 18 year old Muslim Arab Americans	Beliefs and attitudes toward psychotherapy		Therapy described as useful and un-useful Preference for friends and family or spiritual healing Barriers: religion, stigma, and shame, mistrust of MH workers, lack of awareness, lack of access
Straiton et al. (2014) [100]	Norway	Quantitative	15,053	> 18 year old Iraqi immigrants	Rate of use of primary health care services for mental health problems		Rate of GP consultations with psychiatric consultations slightly higher among men
Tomasi et al. (2022) [101]	Australia	Quantitative	1180	> 18 year old Iraqi refugees	Predictors of professional help seeking for mental health problems		Age, psychological distress, presence of disability or long-term illness associated with increased help-seeking Lower financial issues associated with lower help-seeking
Vally et al. (2018) [102]	UAE	Quantitative	114	> 18 year old female undergraduate students	Relationship between both public and self-stigma, and help-seeking attitudes		High public stigma and self-stigma associated with less favorable attitudes to help-seeking
Youssef & Deane (2006) [103]	Australia	Qualitative	35	Age not specified- Arab individuals in Australia	Factors that influence utilization of mental-health services		Barriers: Stigma/shame, confidentiality and trust, family influence, lack of knowledge of services and role of professionals Religious leaders and family important source of help
Zalat et al. (2019) [104]	Egypt	Quantitative	240	M = 28 year working and non-working Egyptian Females	Stigma and attitudes toward seeking psychological help		Social support and personal stigma predict total self-stigma and attitude towards seeking mental health services Less stigmatized views in working females

Help-seeking was operationalized differently across studies and included information on help from professional personnel and formal services, as well as support from informal sources like friends, family, the internet/digital help, and religious faith healers. Since information on the mental health status of participants was not always provided and data focused on hypothetical scenarios, conclusions drawn by many of the articles were therefore based on attitudes towards help-seeking and help-seeking intentions regardless of if individuals ever sought support, rather than actual behaviours.

Overall, measures of help-seeking were heterogeneous across studies. Of the studies that used quantitative measures related to help-seeking outcomes, 21 studies utilised tools developed by the researchers consisting of questionnaires and surveys relying on self-report. Other studies used validated measures related to help-seeking attitudes and behaviours such as the Attitudes Toward Seeking Professional Psychological Help (ATSPPH).

### Quality assessment

According to The Mixed Methods Appraisal Tool (MMAT), the studies included in this review ranged in methodological quality and the supplementary file provides detailed information on the quality of every study. 19 papers *totally met* the specified criteria for qualitative studies (i.e. had “yes” as an answer on all five criteria), and nine papers *partially met* the criteria (i.e. had “yes” as an answer on 2 to 4 of the five criteria). No papers *did not meet* the criteria (i.e. had “yes” as answer on 0 to 1 of the five criteria). Overall quality of papers was good given that appropriate qualitative approaches were adopted and adequate data collection methods such as in-depth interviews and focus groups were used depending on the research questions. The main issue identified was the interpretation of results not always sufficiently substantiated by data. Papers did not always provide quotes from participants to support the interpretations of themes, which is especially important given that many papers performed a thematic analysis. Additionally, few papers provided a clear strategy addressing the subjectivity of the author’s position in terms of data interpretation and hence researcher reflexivity was not sufficiently addressed.

The majority of quantitative descriptive studies (*n* = 35) *partially met,* one study *totally met*, and two studies *did not meet* the specified criteria. In terms of quantitative non-randomized studies, all three included papers *partially met* the specified criteria. The most common problem linked to inconsistency regarding measurements of help-seeking, most studies using self-developed tools to address help-seeking rather than standardised measures. Use of purposive or convenience sampling in most of the studies made it difficult to ensure that participants were representative of the target population. Additionally, description of characteristics of non-responders was not given, making it difficult to generalise results of studies. Use of self-report measures may have also increased risk of biased findings, however anonymous questionnaires could have decreased the effects of social desirability on participant responses. Importantly, many studies did not provide clear information on missing data, and most studies did not consider the role of confounders which could affect the interpretation of results.

Lastly, two mixed methods *partially met* the specified criteria, two studies *did not meet* the criteria, and one study *totally met* the specified criteria. The main consideration is that some studies did not provide an adequate justification for using a mixed methods design or effectively integrate the qualitative and quantitative findings. Divergences between qualitative and quantitative results weren’t addressed in multiple papers. The studies also presented some inconsistencies in addressing specific components of both qualitative and quantitative research. Hence, the overall quality of the empirical work in this area is of a useful research standard, however limitations to presented results should be taken into consideration.

### Outcomes and related themes

#### Help-Seeking behaviours and attitudes

Few studies addressed help-seeking behaviours and included information on actual help-seeking rates and use of support or services (*n* = 11). Many studies provided information on help-seeking attitudes (*n* = 47), however, definitions varied across papers encompassing a range of concepts including help-seeking preferences, perceptions, acceptability, as well as intentions (likelihood/willingness). Papers reported on more than one dimension of help-seeking attitudes.

#### Rate of help-seeking and service utilisation

Despite the need for psychological support, studies generally indicated low rates of service utilisation. Arabs (whether in Arab countries, immigrants, or refugees) were less likely to visit mental health professionals and reluctant to seek help for mental health problems from formal services compared to other non-Arab ethnicities. One study found Arabs immigrants were equally likely as Turks to access specialised services for a common mental disorder, however less likely to use primary care services in relation to psychological distress [59]. Six studies considered associations between factors such as gender, symptomatology, age, and help-seeking behaviours. Papers reported that men used services more than women [44, 96, 100]. Several papers suggested that distress and level of symptomatology, mostly past traumatic events, are associated with increased use of mental health services in contrast to other studies reporting no association [85, 90]. One study mentioned that being older was associated with increased help-seeking [101]. However, another study did not find associations between neither age or sex and help-seeking behaviours [98].

#### Help-seeking perceptions and preferences

Fiftteen studies discussed help-seeking perceptions. Overall, less favourable and fewer positive perceptions were expressed towards use of formal service, and this was true when comparing Arabs to other ethnicities. One study indicated high acceptability and willingness to try an internet-delivered treatment [70]. Three studies with student participants highlighted enthusiastic and positive attitudes towards counselling and professional care [35, 38, 79]. Two studies highlighted mixed views to therapy, in both refugee and immigrant samples [50, 99]. One study found differences based on nationality, whereby Palestinian and Israeli Arabs reported a greater preference to seek mental health treatment for a psychological problem when compared to Kuwaiti and Egyptian Arabs [45], however, another did not find differences based on country of origin [71].

Twenty-three studies described help-seeking preferences and indicated Arabs largely endorsed traditional and informal help sources. Arabs residing in different countries, immigrants, and refugees, were all more likely to use culturally accepted means of help such as friends, family, religion, or self-help. Faith was an important help strategy and talking to or confiding in family members was considered important. Surprisingly, three studies reported a preference for seeking help from mental health professionals and mentioned physicians and GP as first line of contact [71, 75, 93]. Primary care providers in one study did not endorse religion or self-help when identifying informal help-seeking behaviours [52]. Interestingly, other results indicated that Arabs born in Australia were more likely than Arabs born in Algeria, Egypt, Iraq, and Yemen to willingly consult a mental health professional [70]. Suggested reasons included not being able to afford visits to mental health professionals due to high poverty rates for Arabs living in Arab countries, and low levels of mental health literacy compared to Arabs living in Australia.

#### Factors influencing help-seeking attitudes

A total of 21 studies considered factors influencing help-seeking attitudes. Almost all studies considered student and immigrant samples, and the most common factors were mental health literacy, stigma, gender, age, education, religion, acculturation, and immigrant status. Three studies suggested that beliefs and knowledge about mental health and awareness of services predicted favourable help seeking intentions [49, 60, 78]. Only one study found no relationship between beliefs towards mental illness and help-seeking [79]. One study reported that mental health courses had positive effects on attitudes toward seeking professional psychological help [33], suggesting the importance of psychoeducation, mental health literacy, and anti-stigma campaigns.

Eight studies suggested stigma predicted lower help seeking intention and less favourable attitudes. Only one study reported that shame-focused attitudes did not predict help-seeking attitudes [64]. Mixed findings for religion as a predictor of help-seeking among students was indicated. Compared to Druze and Muslims, Christian subjects in one study were higher in interpersonal openness, perceived mental health services as less stigmatising, and were less likely to use traditional healing systems and therefore more likely to seek formal help if needed [46]. Contrastingly, religiosity did not have a significant relationship on help-seeking attitudes in other studies [58, 71].

Nine studies reported that gender predicted help-seeking attitudes, whereby females reported higher help-seeking intentions than men and more positive attitudes. One study considered that females showed higher knowledge of mental illness which could explain higher levels in help-seeking intentions [60], and another study suggested men were more likely to feel stigmatised [35]. Two studies did not find a significance for gender in relation to help-seeking attitudes [64, 71].

Four studies indicated age predicted help-seeking attitudes. Two studies supported that younger respondent had fewer positive attitudes compared to older individuals. Additionally, educational attainment was a predictor of positive attitudes to help-seeking in four studies, one study indicating that first- and second-year students had fewer positive attitudes [47].

Four studies suggested association between acculturation and help-seeking. One study did not [58]. Studies generally reported that higher acculturation impacted attitudes to formal services and one study suggested that the adoption of mainstream culture was associated with semiformal and formal help-seeking [82]. Interestingly, immigrants in one study with greater levels of mainstream acculturation had higher levels and willingness to seek help [53], and another study suggested that greater time in host country predicted more positive attitudes among immigrants [64]. Furthermore, second generation immigrants were more likely to utilize mental health services compared to first generation, who were more likely to use culturally accepted means like friends and family [32].

#### Help-seeking barriers and facilitators

Barriers and facilitators to help-seeking were significantly reported (*n* = 46) and were conceptualised as factors hindering or promoting help-seeking attitudes and behaviours. The majority of findings were reported in relation to formal mental health care rather than informal sources of support.

#### Help-seeking barriers

##### Stigma

More than half the studies (*n* = 34) referred to stigma as a primary obstacle to help-seeking. Both individuals residing in Arab countries and non-Arab countries including refugee and immigrant samples mentioned existing stigma at multiple levels of immediate and larger environments. Refugees experienced stigma, reporting discrimination by mental health professionals in host countries as a barrier to help-seeking, whereas clinicians reported refugee’s avoidance of care due to stigmatisation related to psychiatric diagnosis or treatment.

##### Privacy and confidentiality

To a lesser extent, eight studies reported the issue of privacy and confidentiality as a barrier to seeking help. Overall, individuals worried their confidentiality might be breached or knowledge of their mental health issues might circulate around the community, defecting their image or reputation. Only one study considered electronic mental health services among Arab students in Egypt [80], and another study considered refugees and Lebanese community members in Lebanon [88]. Issues surrounding privacy and confidentiality were otherwise only conveyed by refugee and immigrant samples, however this was less of an issue in host countries compared to their countries of origin [34]. Interestingly, worries about confidentiality were expressed in relation to the use of interpreters in two studies [31, 76], whereby individuals hesitated to share their experiences with professionals in the presence of interpreters describing them as unreliable and non-confidential, especially within a small community of Arabs.

##### Mental health literacy

The second most mentioned barrier was mental health literacy which refers to the knowledge and beliefs about mental illness and available help including recognition, management, and prevention [105]. 28 studies reported lack of awareness regarding where to access mental health services and knowledge on mental health in general across respondents in different countries, professionals, and carers, as well as refugees. This included misattribution of symptoms and causes of mental health, and misconceptions of the role of psychologists. Only one study indicated high levels of mental health literacy regarding knowledge of available professional help [72]. Perceived severity of the problem and ability to recognize need for help, explained preferences to deal with the problem alone. Those who never had a mental health issue preferred self-treatment compared to people who experienced mental health issues before or knew someone who did [67]. Unfamiliarity and beliefs about treatment were also cited as a reason for not seeking help from formal services.

##### Logistical barriers

Seventeen studies referred to logistical barriers which hindered help-seeking. Long wait times for appointments, financial costs, availability of services and staff, and transportation were commonly reported. Arab adults residing in Arab countries, refugees, and immigrants, as well as service providers and family carers reported financial limitations specifically, including issues with initial consultation, continuing care, and medication costs. One quantitative study exceptionally found lower financial issues among refugees to be associated with lower help-seeking [101]. This could be that refugees experiencing financial hardships and other difficulties were receiving support from other social services.

##### Language

Language and communication barriers were reported in ten studies, primarily by refugee populations and Arabs in non-Arabic speaking countries. Service providers working with a refugee population in one study mutually reported language as a barrier [51]. Individuals reported difficulties in approaching professionals due to language barriers finding it challenging to express themselves. Language differences between host communities and the refugees made it tough for individuals to articulate needs and explain problems when speaking to a professional. The use of interpreters facilitating language differences also raised concerns regarding misinterpretation and miscommunication in translation.

##### Cultural barriers

Cultural barriers to help-seeking were mentioned in ten studies. Lack of multicultural competence of professionals and difficulties feeling understood were especially reported by refugees and immigrant minorities in non-Arab countries, whereby individuals expressed a mismatch between needs and available western mental health services. Resorting to alternative ways to getting better that were more culturally appropriate such as religious or traditional healers also influenced the likelihood to seek help from formal services. Interestingly, one study reported cultural norms of enduring difficulties rather than seeking help [92]. One study mentioned that Syrian refugees were more likely to seek help from formal services in Norway compared to Syria due to cultural differences between the two countries and acceptability of treatment [31]. Service providers in one study [41], and refugees in another study [73], mentioned higher prioritisation of daily life challenges and physical health problems as reasons to not seek help.

##### Trust

Ten studies revealed trust as an important barrier. Immigrant and refugee populations in two studies expressed distrust in western mental health care systems [62, 94], and this was supported by one study with mental health professionals working with refugees [51]. Issues surrounding mistrust and lack of confidence in competency and transparency of professionals were revealed, mostly in refugee and immigrant samples. Participants felt reluctant to disclose personal information having limited trust in the quality of services.

#### Help-seeking facilitators

Out of the 74 included studies, facilitators to help-seeking were significantly less reported (*n* = 4). One study mentioned increasing societal and family awareness, external support, encouragement of seeking support, the want to change, and online services, as facilitators to help seeking [88]. Professionals in two qualitative studies working in Arab and non-Arab countries expressed that a shared culture between client and therapist is an important facilitator to treatment [63] and the quality of doctor patient relationship influences likelihood of seeking or continuing treatment [37]. Refugees in one quantitative study established that feelings of connectedness with the host country and social integration play a role in promoting help seeking from a GP [65].

#### Other factors

Some studies reported mixed findings for the role of religion, and family and community, in relation to facilitating or hindering help-seeking. Two studies regarded religion as a barrier, and this was expressed by carers of people with mental illness and psychiatrists [41, 99]. Interestingly, being from a certain religion compared to others either facilitated or hindered help-seeking. Refugees and immigrants in two studies indicated certain interpretations in Islam perceived mental illness as a form of punishment from God and therefore considered seeking treatment to be against God’s will [83, 94]. Seeking help from religious healers was less stigmatising and more confidential. However, in one study, Arabs described how faith facilitated help-seeking and described the convergence between Muslim values and help-seeking [39].

The role of family and community members was expressed as a barrier to help-seeking in two studies, with individuals expressing discouragement from family members to seek psychiatric help [66, 103]. Conversely, two studies described how positive influence from social networks facilitated help-seeking [75, 77]. Three studies identified the role of family and community support as being both facilitating and hindering help seeking [40, 63, 88]. One study found no association between parental support and help-seeking attitudes [79].

## Discussion

To our knowledge, this systematic review was first to explore experiences of mental health help-seeking among Arab populations globally. The 74 identified studies demonstrated that Arabs across countries portrayed similarities in help-seeking behaviours and attitudes, identifying common barriers and facilitators in their experiences. Participants largely varied in age, gender, occupation, geographic location or residence, and range of mental health problems.

Results indicate common mental health difficulties among Arabs across countries, however, rates of help-seeking are generally low and individuals use formal services less than other non-Arab ethnicities. This is consistent with findings from a systematic review on Filipino ethnic minorities across countries, where high levels of psychological distress but low utilisation of mental health services were found [11], and comparable to Chinese, South Asian, and Southeast Asian immigrants [106], African American and Hispanic populations [107], and other ethnic minorities [108]. There is a lack of data regarding factors influencing help-seeking behaviours. Mixed findings associating higher distress levels and symptomatology and increased help-seeking were found. Research generally reports on levels of distress in relation to help-seeking attitudes rather than behaviours, however, clinical characteristics partially explain the type of help-seeking in Chinese populations [109]. Older people and males were more likely to use services in this review. These factors are also reported as determinants to help-seeking in the literature [110, 111]. People might perhaps not seek help in a timely way when symptoms emerge, or younger individuals might start with lower symptom levels therefore not seeking help at a young age. However, as people get older and remaining difficulties increase in severity, they might be more likely to seek help.

Furthermore, Arabs across countries have negative attitudes towards formal help-seeking, reluctance to seek help, and preferred informal sources of psychological support. These findings are consistent with help-seeking attitudes in Filipino [11], Asian American [112], and Indian ethnic minority populations [113]. Differences in help-seeking attitudes among Arabs from different countries is not thoroughly addressed in existing literature, and only one study found differences [45], while another did not [47]. Therefore, future research should consider how a country of origin may influence help-seeking attitudes. Contextual influences on Arab help-seeking attitudes should also be considered in future research, as only two studies highlighted that Arabs in non-Arab countries show higher willingness and acceptability to visit a mental health professional overseas in contrast to their native country [31, 70]. However, included studies generally suggested that higher acculturation and more years spent in host countries by refugees and immigrants predicted favourable attitudes to help-seeking. A study with similar results indicated higher levels of acculturation meant more positive attitudes toward seeking help among Vietnamese Americans [114]. This could explain exposure and acculturation to cultures that are more tolerant of mental health stigma, probably influencing more favourable attitudes to formal services.

The influence of stigma and gender on help-seeking attitudes was highlighted in included studies. Conceptualizations of mental illness and societal stigma influenced willingness and intentions to seek help and predicted fewer positive attitudes to help-seeking. This is consistent among European ethnicities [115]. Gender predicted help-seeking attitudes and although men had higher rates of service use, they endorsed less favourable attitudes to help-seeking compared to women. This contrasts with studies suggesting attitudinal barriers as predictive of service utilisation [116], where men displayed less favourable attitudes and lower help-seeking behaviours [117]. It is unclear why high intentions to seek help among women did not reflect higher help-seeking behaviours, but this may suggest differences in barriers to help-seeking between men and women. Gender differences in help-seeking attitudes compare to other findings [118], and a previous systematic review suggested conformity to traditional masculine gender norms impacts attitudes related to help-seeking and may explain less favourable attitudes among men [119]. It might be that Arab men are less likely to seek informal support from friends and family due to stigma and fear of how they may be perceived as weak and more likely to utilise formal services. However, results from one study highlight that women and men have largely similar attitudes about informal help seeking, and only differ in attitudes towards formal help-seeking [120].

Although other factors affecting help-seeking attitudes such as mental health literacy, age, educational attainment, and religion were not among the most cited in the review, some interesting points can be made. Mental health literacy predicted more positive attitudes to help-seeking and this is comparable to other populations [121], and to Arab student’s attitudes in this review. Only one study contrastingly documented pessimistic attitudes and high levels of mental health literacy among students in this review [72]. Studies simultaneously indicated that older age and higher educational attainment were associated with more positive attitudes to help-seeking. These findings are contradictory, suggesting other factors specific to the populations sampled in the included studies may explain these observations.

Prominent barrier themes to help-seeking in this review are consistent with commonly reported obstacles to accessing care and seeking psychological support among Asian Americans [122], Asian and Latin American adolescents [123], and refugees in high income countries [124]. Stigmatisation of Arab individuals by society and one’s own stigma toward mental health treatment remained a major barrier to mental health treatment. Stigma is repeatedly reported as a significant barrier to help-seeking in previous reviews [11, 125, 126], and among ethnic minority populations and refugees across regions [127, 128]. Fear of cultural and societal stigma stemmed from individuals possibly being treated differently and marginalised because of labels, avoided in social situations, or isolated by community members. Mental health literacy was the second most prominent barrier to help-seeking, and misinformation or a lack of information about mental health and available services was also a barrier in a review on East Asian immigrants [129]. Practical barriers to the use of mental health services like accessibility and financial constraints also hindered help-seeking in similarity to reported barriers among ethnically diverse and impoverished women [130] and black and ethnic minority communities [131]. The deterrent roles of language differences, trust, privacy, and cultural differences were reported less, however, they were especially applicable to immigrant and refugee populations. Similar findings have been reported in non-Arab refugee and immigrant samples suggesting negative experiences in host countries may discourage individuals from seeking help [132].

Facilitators to help-seeking were much less reported, precluding many conclusions from being drawn. Facilitators including social integration in host countries and culturally competent staff were expressed by refugees and immigrants as promoting treatment. Most literature only reports barriers to treatment among refugees. However, one study considered use of services aiming to facilitate settlement reporting a positive association between inter-ethnic networks and use of those services [133]. It is difficult to conclude a certain association between refugee integration and mental health help-seeking, but literature suggests the importance of social integration on mental health [134].

One study in the review reported that quality of doctor-patient relationship influences the likelihood of seeking and continuing treatment [37]. A systematic review reported that certain characteristics and behaviours of professionals such as empathy and trustworthiness helped patients with post-traumatic stress disorder (PTSD) feel comfortable, and competent caring providers were key in initiating help-seeking and continuing care [135]. Immigrant and refugee women with postpartum depression (PPD) also reported health care relationship as a critical determinant to seek and accept help [136]. More research is needed to understand facilitators to help-seeking for Arabs, especially among non-refugee and non-immigrant Arab samples. Consideration of adults who sought help from services would aid our understanding of real-life factors leading to seeking help.

Conflicting findings on factors that served both as facilitators and barriers to Arab help-seeking were concluded upon synthesis of the studies, specifically: (1) Role of family and community; (2) Religion.

O’Mahony et al. [136] similarly reported social networks as being supportive or non-supportive with effects on psychological health and well-being, and perceptions of need for care within close networks (i.e., told by family/friends to seek professional help) were reported as significant predictors of service use among Latinos in the United States [137]. A study documented spouses and children as key reasons patients were motivated to engage in treatment [135], and social support from family and friends played an important role in professional help-seeking for suicidality [138]. Contrastingly, lower perceived social support from family was a significant predictor of positive help-seeking attitudes and greater help-seeking behaviour among Mexican American students [139]. This could be individuals feeling an increased need to seek out help if they sense a lack of support from social networks around them.

Religion is not consistently reported in the literature and one study documented religion and spirituality as barriers to mental health help-seeking among African American youth [140]. Greater distrust towards healthcare systems and concern surrounding social stigma associated with seeking treatment was expressed among religious clients in one study [141]. Literature suggests that mental health attributions and causal beliefs regarding the aetiology of mental illness influence help-seeking behaviours [142], whereby ethnic minorities are more likely to mention spirituality causes to mental illness [143]. This might explain why individuals are less likely to access formal services and rely on faith-based healers and spiritual treatments.

### Strengths and limitation

This review was first to assess a wide range of mental health help-seeking related outcomes in Arab populations around the world. The robust systematic search strategy aimed to strike a balance between precision and sensitivity, using optimal database combination. We chose to preserve sensitivity and comprehensiveness to capture relevant published literature. We adhered to the PRISMA guidelines to conduct and report systematic reviews, and independent reviewers were included in the screening stage. The mixed methods appraisal tool (MMAT) utilised in previous systematic reviews and with demonstrated validity and reliability was chosen to assess quality of included studies.

Several limitations also need to be considered. Firstly, participants of included studies had to be 18 years of age or older, meaning studies with elderly populations were included, and help-seeking for younger and older adults may differ due to generational differences [32]. Additionally, needs of refugee populations may differ from immigrants and therefore require alternative help-seeking pathways. Moreover, this review prioritised the overinclusion of studies for a comprehensive picture of the existing evidence regarding mental health help-seeking in Arabs. As a result, the analysis included low quality studies, and this affected the interpretation of the findings. Additionally, most quantitative studies were cross-sectional, making it difficult to determine temporal directions of associations which is necessary to establish causality. Many of the included studies were low in external validity using convenience sampling, making it difficult to generalise results. A crucial limitation pertained to differences in help-seeking measures and definitions used, making comparison of results difficult. The heterogeneous nature of studies did not allow for a meta-synthesis. The measures were western-based inventories, and therefore may not have suited cross-cultural research on Arab participants. Lastly, the search was restricted to the English language. However, no appropriate papers in a non-English language were found during the soft search prior to the systematic search and therefore this is unlikely to have been a considerable limitation.

### Implications

This review highlights gaps in the literature proposing several implications for research, practice, and policy. Although help-seeking behaviours and attitudes are separate constructs, many studies in this review reported help-seeking intentions and attitudes as actual behaviours. Hence, future research needs to further operationalize these definitions. Most data reported on help-seeking attitudes and intentions rather than actual behaviours, therefore, future research should focus on actual utilisation rates of services to gain better understanding of factors that influence help-seeking behaviours. Majority of studies discussed help-seeking barriers rather than facilitators and a focus on factors promoting help-seeking is needed in future research. Results showed that help-seeking is more problematic for Arabs in their native countries and that acculturation in host countries allows for better engagement with services and help sources. Therefore, increasing mental health literacy to reduce stigma through psychoeducation and anti-stigma campaigns could be employed to encourage help-seeking. Interventions targeting friends and family could be useful in changing help-seeking attitudes and behaviours, as they might motivate and encourage individuals to seek help from mental health services. The importance of culturally informed interventions and the need for Black, Asian and minority ethnic (BAME) representation amongst healthcare professionals has been documented in various studies [129, 144, 145]. Such efforts may provide solutions in overcoming barriers to help-seeking among Arabs, allowing individuals to receive treatment from professionals they can relate to on an ethnic and cultural basis. Finally, mental health legislations should focus on areas such as prevention, early identification and intervention, and integrated care and recovery, for Arab populations in different countries. Policies designed to address the status of Arab immigrants and refugees in order to recognize, protect, and facilitate their ability to seek mental health support is especially needed.

## Conclusion

The findings of this review highlight that help-seeking among Arab populations globally, is influenced by cultural and to some extent by religious values, mental health literacy, and stigma.

Despite expectations of the role of migration and acculturation that might influence help-seeking behaviours and attitudes, only few findings showed that the process of acculturation in host countries can increase the likelihood and acceptability of help-seeking. There still remains a high proportion of those in need of mental health support who may not access services, as individuals fundamentally hold onto cultural beliefs regardless of where they live. Additional logistical barriers that influence help-seeking in host countries among immigrated individuals include factors related to language and culture.

This review highlights the importance of increasing mental health literacy through psychoeducation and anti-stigma campaigns, developing culturally appropriate and accessible clinical interventions, and ensuring that clinicians are representative of the populations they serve.

## Supplementary Information

**Additional file 1: Supplementary file 1.** Quality assessment of included studies.

**Additional file 2: Supplementary file 2.** Search strategy.

## Data Availability

The datasets used and/or analysed during the current study available from the corresponding author on reasonable request. Contact Madiha.shaikh@ucl.ac.uk https://www.cabidigitallibrary.org/doi/10.1079/searchRxiv.2022.00059
