# Supplementary material for: Understanding experiences of mental health help-seeking in Arab populations around the world: a systematic review and narrative synthesis
Source: BMC Psychiatry. 2023 May 9;23:324. doi: 10.1186/s12888-023-04827-4 (PMC10170733; doi:10.1186/s12888-023-04827-4)
Supplement: Supplementary file 1 — Additional file 1: Supplementary file 1. Quality assessment of included studies. [file 12888_2023_4827_MOESM1_ESM.docx]

Supplementary file 1: Quality of included studies

**Table 1. MMAT Assessment of Qualitative Studies**

| **MMAT Criteria** | **Study** | | | | | |
| --- | --- | --- | --- | --- | --- | --- |
|  | **Aarethun et al. (2021)**  [(31)](#bookmark=id.ihv636) | **Al-Busaidi (2010)**  [(37)](#bookmark=id.3fwokq0) | **Al-Darmaki et al. (2016)**  [(38)](#bookmark=id.1v1yuxt) | **Al-Dousari & Prior (2020)**  [(39)](#bookmark=id.4f1mdlm) | **Alhomaizi et al. (2018)**  ([40)](#bookmark=id.2u6wntf) | **AlLaham et al. (2020)**  ([48)](#bookmark=id.2lwamvv) |
| **Are there clear research questions?** | Yes | Yes | Yes | Yes | Yes | Yes |
| **Do the collected data allow to address the research questions?** | Yes | Yes | Yes | Yes | Yes | Yes |
| **Is the qualitative approach appropriate to answer the research question?** | Yes | Yes | Yes | Yes | Yes | Yes |
| **Are the qualitative data collection methods adequate to address the research question?** | Yes | Yes | Yes | Yes | Yes | Yes |
| **Are the findings adequately derived from the data?** | Yes | Yes | Yes | Yes | Yes | Yes |
| **Is the interpretation of results sufficiently substantiated by data?** | Yes | Can't tell | No | Can't tell | Yes | Yes |
| **Is there coherence between qualitative data sources, collection, analysis and interpretation?** | Yes | Yes | Yes | Yes | Yes | Yes |
| **Quality** | Totally met | Partially met | Partially met | Partially met | Totally met | Totally met |
| *Totally met = “yes” on the five criteria, Partially met = “yes” on 2 to 4 of the five criteria, Not met = “yes” on 0 to 1 of the five criteria* | | | | | | |

**Table 1. Continued**

| **MMAT Criteria** | **Study** | | | | | |
| --- | --- | --- | --- | --- | --- | --- |
|  | **Al-Roubaiy et al. (2017)**  [(50)](#bookmark=id.3l18frh) | **Al-Soleiti et al (2021)**  ([51)](#bookmark=id.206ipza) | **Ayalon et al. (2015)**  ([52)](#bookmark=id.4k668n3) | **Bawadi et al. (2022)**  ([55)](#bookmark=id.3ygebqi) | **Dogan et al. (2019)**  ([57)](#bookmark=id.sqyw64) | **Gundel et al. (2016)**  [(62)](#bookmark=id.1664s55) |
| **Are there clear research questions?** | Yes | Yes | Yes | Yes | Yes | Yes |
| **Do the collected data allow to address the research questions?** | Yes | Yes | Yes | Yes | Yes | Yes |
| **Is the qualitative approach appropriate to answer the research question?** | Yes | Yes | Yes | Yes | Yes | Yes |
| **Are the qualitative data collection methods adequate to address the research question?** | Yes | Yes | Yes | Yes | Yes | Yes |
| **Are the findings adequately derived from the data?** | Yes | Yes | Yes | Can't tell | Yes | Yes |
| **Is the interpretation of results sufficiently substantiated by data?** | Yes | No | Yes | Yes | Yes | Yes |
| **Is there coherence between qualitative data sources, collection, analysis and interpretation?** | Yes | Yes | Yes | Yes | Yes | Yes |
| **Quality** | Totally met | Partially met | Totally met | Partially met | Totally met | Totally met |
| *Totally met = “yes” on the five criteria, Partially met = “yes” on 2 to 4 of the five criteria, Not met = “yes” on 0 to 1 of the five criteria* | | | | | | |

**Table 1. Continued**

| **MMAT Criteria** | **Study** | | | | | |
| --- | --- | --- | --- | --- | --- | --- |
|  | **Habhab (2018)**  [(63)](#bookmark=id.3q5sasy) | **Hasan & Musleh (2017)**  [(66)](#bookmark=id.34g0dwd) | **Khatib & Abo-Rass (2021)**  ([72)](#bookmark=id.1x0gk37) | **Kiselev et al. (2020)**  ([73)](#bookmark=id.4h042r0) | **Linney et al. (2020)**  [(75)](#bookmark=id.1baon6m) | **Loewenthal et al.**  ([76)](#bookmark=id.3vac5uf) |
| **Are there clear research questions?** | Yes | Yes | Yes | Yes | Yes | Yes |
| **Do the collected data allow to address the research questions?** | Yes | Yes | Yes | Yes | Yes | Yes |
| **Is the qualitative approach appropriate to answer the research question?** | Yes | Yes | Yes | Yes | Yes | Yes |
| **Are the qualitative data collection methods adequate to address the research question?** | Yes | Yes | Yes | Yes | Yes | Yes |
| **Are the findings adequately derived from the data?** | Yes | Yes | Yes | Yes | Yes | Yes |
| **Is the interpretation of results sufficiently substantiated by data?** | Yes | Yes | No | Yes | Yes | No |
| **Is there coherence between qualitative data sources, collection, analysis and interpretation?** | Yes | Yes | Yes | Yes | Yes | Yes |
| **Quality** | Totally met | Totally met | Partially met | Totally met | Totally met | Partially met |
| *Totally met = “yes” on the five criteria, Partially met = “yes” on 2 to 4 of the five criteria, Not met = “yes” on 0 to 1 of the five criteria* | | | | | | |

| **MMAT Criteria** | **Study** | | | | | |
| --- | --- | --- | --- | --- | --- | --- |
|  | **McKell et al. (2017)**  [(83)](#bookmark=id.1302m92) | **Mahajan et al. (2022)**  ([77)](#bookmark=id.2afmg28) | **Molsa et al. (2010)**  ([84)](#bookmark=id.3mzq4wv) | **Noorwali et al. (2022)**  [(88)](#bookmark=id.1gf8i83) | **Noubani et al. (2020)**  ([89)](#bookmark=id.40ew0vw) | **Piwowarczyk et al. (2014)**  [(91)](#bookmark=id.upglbi) |
| **Are there clear research questions?** | Yes | Yes | Yes | Yes | Yes | Yes |
| **Do the collected data allow to address the research questions?** | Yes | Yes | Yes | Yes | Yes | Yes |
| **Is the qualitative approach appropriate to answer the research question?** | Yes | Yes | Yes | Yes | Yes | Yes |
| **Are the qualitative data collection methods adequate to address the research question?** | Yes | Yes | Yes | Yes | Yes | Yes |
| **Are the findings adequately derived from the data?** | Yes | Yes | Yes | Yes | Yes | Yes |
| **Is the interpretation of results sufficiently substantiated by data?** | Yes | No | Yes | No | Yes | Yes |
| **Is there coherence between qualitative data sources, collection, analysis and interpretation?** | Yes | Yes | Yes | Yes | Yes | Yes |
| **Quality** | Totally met | Partially met | Totally met | Partially met | Totally met | Totally met |
| *Totally met = “yes” on the five criteria, Partially met = “yes” on 2 to 4 of the five criteria, Not met = “yes” on 0 to 1 of the five criteria* | | | | | | |

**Table 1. Continued**

**Table 1. Continued**

| **MMAT Criteria** | **Study** | | | |
| --- | --- | --- | --- | --- |
|  | **Rae (2014)**  [(92)](#bookmark=id.3ep43zb) | **Said et al. (2021)**  ([94)](#bookmark=id.4du1wux) | **Smith (2011)**  [(99)](#bookmark=id.meukdy) | **Youssef & Deane (2006)**  [(103)](#bookmark=id.2koq656) |
| **Are there clear research questions?** | Yes | Yes | Yes | Yes |
| **Do the collected data allow to address the research questions?** | Yes | Yes | Yes | Yes |
| **Is the qualitative approach appropriate to answer the research question?** | Yes | Yes | Yes | Yes |
| **Are the qualitative data collection methods adequate to address the research question?** | Yes | Yes | Yes | Yes |
| **Are the findings adequately derived from the data?** | Yes | Yes | Yes | Yes |
| **Is the interpretation of results sufficiently substantiated by data?** | Yes | Yes | Yes | Yes |
| **Is there coherence between qualitative data sources, collection, analysis and interpretation?** | Yes | Yes | Yes | Yes |
| **Quality** | Totally met | Totally met | Totally met | Totally met |
| *Totally met = “yes” on the five criteria, Partially met = “yes” on 2 to 4 of the five criteria, Not met = “yes” on 0 to 1 of the five criteria* | | | | |

**Table 2: MMAT Assessment of Quantitative Descriptive Studies**

| **MMAT Criteria** | **Study** | | | | | |
| --- | --- | --- | --- | --- | --- | --- |
|  | **Abubotain (2021)**  ([32)](#bookmark=id.32hioqz) | **Al Ali et al. (2017)**  ([36)](#bookmark=id.vx1227) | **Alissa (2021)**  [(42)](#bookmark=id.3tbugp1) | **Alkhayat-Hatahet (2021)**  ([43)](#bookmark=id.28h4qwu) | **Al-Krenawi (2002)**  ([44)](#bookmark=id.nmf14n) | **Al-Krenawi et al. (2009)**  [(45)](#bookmark=id.37m2jsg) |
| **Are there clear research questions?** | Yes | Yes | Yes | Yes | Yes | Yes |
| **Do the collected data allow to address the research questions?** | Yes | Yes | Yes | Yes | Yes | Yes |
| **Is the sampling strategy relevant to address the research question?** | Yes | Yes | Can't tell | Yes | Yes | Yes |
| **Is the sample representative of the target population?** | No | No | Yes | No | Can't tell | No |
| **Are the measurements appropriate?** | Yes | Yes | Can't tell | Yes | Can't tell | Yes |
| **Is the risk of nonresponse bias low?** | Can't tell | Can't tell | Can't tell | Can't tell | Can't tell | Can't tell |
| **Is the statistical analysis appropriate to answer the research question?** | Yes | Yes | Yes | Yes | Can't tell | Yes |
| **Quality** | Partially met | Partially met | Partially met | Partially met | Did not meet | Partially met |
| *Totally met = “yes” on the five criteria, Partially met = “yes” on 2 to 4 of the five criteria, Not met = “yes” on 0 to 1 of the five criteria* | | | | | | |

**Table 2. Continued**

| **MMAT Criteria** | **Study** | | | | | |
| --- | --- | --- | --- | --- | --- | --- |
|  | **Al-Krenawi & Graham (2011)**  [(46)](#bookmark=id.1mrcu09) | **Al-Krenawi et al. (2004)**  [(47)](#bookmark=id.46r0co2) | **Aloud & Rathur (2009)**  [(49)](#bookmark=id.111kx3o) | **Balesh et al. (2018)**  [(53)](#bookmark=id.28h4qwu) | **Bashir et al. (2020)**  ([54)](#bookmark=id.1egqt2p) | **Castaneda et al. (2020)**  [(56)](#bookmark=id.2dlolyb) |
| **Are there clear research questions?** | Yes | Yes | Yes | Yes | Yes | Yes |
| **Do the collected data allow to address the research questions?** | Yes | Yes | Yes | Yes | Yes | Yes |
| **Is the sampling strategy relevant to address the research question?** | Yes | Yes | Yes | Yes | Yes | Yes |
| **Is the sample representative of the target population?** | Can't tell | No | Yes | Can't tell | Yes | Yes |
| **Are the measurements appropriate?** | Yes | Yes | Yes | Yes | Yes | Yes |
| **Is the risk of nonresponse bias low?** | Can’t tell | Can't tell | Can’t tell | Can't tell | Can’t tell | Can't tell |
| **Is the statistical analysis appropriate to answer the research question?** | Yes | Yes | Yes | Yes | Yes | Yes |
| **Quality** | Partially met | Partially met | Partially met | Partially met | Partially met | Partially met |
| *Totally met = “yes” on the five criteria, Partially met = “yes” on 2 to 4 of the five criteria, Not met = “yes” on 0 to 1 of the five criteria* | | | | | | |

**Table 2. Continued**

| **MMAT Criteria** | **Study** | | | | | | |
| --- | --- | --- | --- | --- | --- | --- | --- |
|  | **Elghoroury (2017)**  [(58)](#bookmark=id.3cqmetx) | **Fassaert et al. (2009)**  ([59](#bookmark=id.1rvwp1q)) | **Fekih-Romdhane et al. (2021)**  [(60)](#bookmark=id.4bvk7pj) | **Fuhr et al. (2020)**  ([61)](#bookmark=id.2r0uhxc) | **Hamid & Furnham (2013)**  [(64)](#bookmark=id.25b2l0r) | **Harris et al. (2021)**  ([65)](#bookmark=id.kgcv8k) | **Kamel et al. (2021)**  [(67)](#bookmark=id.1jlao46) |
| **Are there clear research questions?** | Yes | Yes | Yes | Yes | Yes | Yes | Yes |
| **Do the collected data allow to address the research questions?** | Yes | Yes | Yes | Yes | Yes | Yes | Yes |
| **Is the sampling strategy relevant to address the research question?** | Yes | Yes | Can't tell | Yes | Can't tell | Yes | Can’t tell |
| **Is the sample representative of the target population?** | Yes | Yes | No | No | Can't tell | Yes | Can't tell |
| **Are the measurements appropriate?** | Yes | Yes | Yes | Yes | Yes | Yes | Can’t tell |
| **Is the risk of nonresponse bias low?** | Can’t tell | Yes | Yes | Can't tell | Can't tell | Can't tell | Can't tell |
| **Is the statistical analysis appropriate to answer the research question?** | Yes | Yes | Yes | Can't tell | Yes | Can't tell | Yes |
| **Quality** | Partially met | Totally met | Partially met | Partially met | Partially met | Partially met | Did not meet |
| *Totally met = “yes” on the five criteria, Partially met = “yes” on 2 to 4 of the five criteria, Not met = “yes” on 0 to 1 of the five criteria* | | | | | | | |

**Table 2. Continued**

| **MMAT Criteria** | **Study** | | | | | |
| --- | --- | --- | --- | --- | --- | --- |
|  | **Karam et al. (2018)**  [(69)](#bookmark=id.2iq8gzs) | **Kayrouz et al. (2015)**  [(71)](#bookmark=id.3hv69ve) | **Kayrouz et al. (2018)**  ([70)](#bookmark=id.xvir7l) | **Levav et al. (2007)**  ([74)](#bookmark=id.2w5ecyt) | **Mahmoud (2018)**  [(78)](#bookmark=id.pkwqa1) | **Mahsoon et al. (2020)**  ([79)](#bookmark=id.39kk8xu) |
| **Are there clear research questions?** | Yes | Yes | Yes | Yes | Yes | Yes |
| **Do the collected data allow to address the research questions?** | Yes | Yes | Yes | Yes | Yes | Yes |
| **Is the sampling strategy relevant to address the research question?** | Can't tell | Yes | Yes | Yes | Yes | Yes |
| **Is the sample representative of the target population?** | Yes | No | No | No | Yes | No |
| **Are the measurements appropriate?** | Yes | Yes | Yes | Yes | Can't tell | Yes |
| **Is the risk of nonresponse bias low?** | Can't tell | Can't tell | Can't tell | Yes | Can't tell | Can't tell |
| **Is the statistical analysis appropriate to answer the research question?** | Yes | Yes | Yes | Yes | Yes | Yes |
| **Quality** | Partially met | Partially met | Partially met | Partially met | Partially met | Partially met |
| *Totally met = “yes” on the five criteria, Partially met = “yes” on 2 to 4 of the five criteria, Not met = “yes” on 0 to 1 of the five criteria* | | | | | | |

**Table 2. Continued**

| **MMAT Criteria** | **Study** | | | | | |
| --- | --- | --- | --- | --- | --- | --- |
|  | **Mamdouh et al. (2022)**  ([80)](#bookmark=id.1opuj5n) | **Markova et al. (2020)**  ([82)](#bookmark=id.2nusc19) | **Molsa et al. (2019)**  ([85)](#bookmark=id.2250f4o) | **Mond et al. (2021)**  [(86)](#bookmark=id.haapch) | **Nazzal (2015)**  [(87)](#bookmark=id.319y80a) | **Palgi et al. (2011)**  ([90)](#bookmark=id.2fk6b3p) |
| **Are there clear research questions?** | Yes | Yes | Yes | Yes | Yes | Yes |
| **Do the collected data allow to address the research questions?** | Yes | Yes | Yes | Yes | Yes | Yes |
| **Is the sampling strategy relevant to address the research question?** | Yes | Yes | Yes | Yes | Can't tell | Yes |
| **Is the sample representative of the target population?** | No | No | Yes | Yes | No | Can't tell |
| **Are the measurements appropriate?** | Can't tell | Yes | Yes | Yes | Yes | Yes |
| **Is the risk of nonresponse bias low?** | Can't tell | Can't tell | Can't tell | Can't tell | Can't tell | Can't tell |
| **Is the statistical analysis appropriate to answer the research question?** | Yes | Yes | Can't tell | Yes | Yes | Yes |
| **Quality** | Partially met | Partially met | Partially met | Partially met | Partially met | Partially met |
| *Totally met = “yes” on the five criteria, Partially met = “yes” on 2 to 4 of the five criteria, Not met = “yes” on 0 to 1 of the five criteria* | | | | | | |

**Table 2. Continued**

| **MMAT Criteria** | **Study** | | | | | | |
| --- | --- | --- | --- | --- | --- | --- | --- |
|  | **Rakhawy (2010)**  [(93)](#bookmark=id.1tuee74) | **Schlechter et al. (2021)**  [(95)](#bookmark=id.2szc72q) | **Schubert et al. (2019)**  [(96)](#bookmark=id.184mhaj) | **Shechtman et al. (2018)**  [(97)](#bookmark=id.3s49zyc) | **Slewa-Younan et al. (2015)**  [(98)](#bookmark=id.279ka65) | **Straiton et al. (2014)**  ([100)](#bookmark=id.36ei31r) | **Vally et al. (2018)**  [(102)](#bookmark=id.45jfvxd) |
| **Are there clear research questions?** | Yes | Yes | Yes | Yes | Yes | Yes | Yes |
| **Do the collected data allow to address the research questions?** | Yes | Yes | Yes | Yes | Yes | Yes | Yes |
| **Is the sampling strategy relevant to address the research question?** | Can't tell | Can't tell | Yes | Can’t tell | Can't tell | Yes | Yes |
| **Is the sample representative of the target population?** | No | Can't tell | Yes | No | Yes | Yes | No |
| **Are the measurements appropriate?** | Yes | Yes | Yes | Yes | Can't tell | Yes | Yes |
| **Is the risk of nonresponse bias low?** | Can't tell | Can't tell | Can't tell | Can't tell | Can't tell | Can’t tell | Can't tell |
| **Is the statistical analysis appropriate to answer the research question?** | Yes | Yes | Yes | Yes | Yes | Yes | Yes |
| **Quality** | Partially met | Partially met | Partially met | Partially met | Partially met | Partially met | Partially met |
| *Totally met = “yes” on the five criteria, Partially met = “yes” on 2 to 4 of the five criteria, Not met = “yes” on 0 to 1 of the five criteria* | | | | | | | |

**Table 3: MMAT Assessment of Quantitative Non-Randomized Studies**

| **MMAT Criteria** | **Study** | | |
| --- | --- | --- | --- |
|  | **Abuhammad & Hamaideh (2022)**  ([33)](#bookmark=id.1hmsyys) | **Tomasi et al. (2022)**  ([101)](#bookmark=id.1ljsd9k) | **Zalat et al. (2019)**  [(104)](#bookmark=id.zu0gcz) |
| **Are there clear research questions?** | Yes | Yes | Yes |
| **Do the collected data allow to address the research questions?** | Yes | Yes | Yes |
| **Are the participants representative of the target population?** | Yes | Yes | No |
| **Are measurements appropriate regarding both the outcome and intervention (or exposure)?** | Yes | Yes | Yes |
| **Are there complete outcome data?** | Can't tell | Can't tell | Can't tell |
| **Are the confounders accounted for in the design and analysis?** | No | No | No |
| **During the study period, is the intervention administered (or exposure occurred) as intended?** | Yes | Yes | Yes |
| **Quality** | Partially met | Partially met | Partially met |
| *Totally met = “yes” on the five criteria, Partially met = “yes” on 2 to 4 of the five criteria, Not met = “yes” on 0 to 1 of the five criteria* | | | |

**Table 4: MMAT Assessment of Mixed Methods Studies**

| **MMAT Criteria** | **Study** | | | | |
| --- | --- | --- | --- | --- | --- |
|  | **Ahmed et al. (2017)**  ([34)](#bookmark=id.41mghml) | **Alajlan (2016)**  [(35)](#bookmark=id.2grqrue) | **Ali & Agyapong (2016)**  ([36)](#bookmark=id.vx1227) | **Karadag et al. (2021)**  [(68)](#bookmark=id.43ky6rz) | **Markova & Sandal (2016)**  ([81)](#bookmark=id.48pi1tg) |
| **Are there clear research questions?** | Yes | Yes | Yes | Yes | Yes |
| **Do the collected data allow to address the research questions?** | Yes | Yes | Yes | Yes | Yes |
| **Is there an adequate rationale for using a mixed methods design to address the research question?** | No | Yes | No | Yes | Yes |
| **Are the different components of the study effectively integrated to answer the research question?** | No | Yes | Yes | No | Yes |
| **Are the outputs of the integration of qualitative and quantitative components adequately interpreted?** | No | Yes | Yes | No | Yes |
| **Are divergences and inconsistencies between quantitative and qualitative results adequately addressed?** | No | Yes | Can't tell | No | No |
| **Do the different components of the study adhere to the quality criteria of each tradition of the methods involved?** | No | Yes | No | No | No |
| **Quality** | Did not meet | Totally met | Partially met | Did not meet | Partially met |
| *Totally met = “yes” on the five criteria, Partially met = “yes” on 2 to 4 of the five criteria, Not met = “yes” on 0 to 1 of the five criteria* | | | | | |
