# Supplementary material for: Understanding experiences of mental health help-seeking in Arab populations around the world: a systematic review and narrative synthesis
Source: BMC Psychiatry. 2023 May 9;23:324. doi: 10.1186/s12888-023-04827-4 (PMC10170733; doi:10.1186/s12888-023-04827-4)
Supplement: Supplementary file 2 — Additional file 2: Supplementary file 2. Search strategy. [file 12888_2023_4827_MOESM2_ESM.docx]

| Concept 1: Mental Health | Concept 2: Arab | Concept 3: Help Seeking | Concept 4: Experiences and Behaviors |
| --- | --- | --- | --- |
| - exp Mental Disorders/  - Mental Health/  - Mental health.ti,ab.  - (Mental adj3 (wellbeing or well being)).ti,ab.  - Mental hygiene.ti,ab.  - mental disorder*.ti,ab.  - mental illness*.ti,ab.  - ((psychologic* or emotional) adj3 distress*).ti,ab. | -middle east/ or bahrain/ or iraq/ or jordan/ or kuwait/ or lebanon/ or oman/ or qatar/ or saudi arabia/ or syria/ or united arab emirates/ or yemen/  -Arabs/  -exp africa, northern/ or djibouti/ or somalia/ or sudan/ or mauritania/  -Comoros/  -Middle East.ti,ab.  -Arab*.ti,ab.  -Bahrain.ti,ab.  -Iraq.ti,ab.  -Qatar.ti,ab.  -Egypt.ti,ab.  -Palsetine.ti,ab.  -Lebanon.ti,ab.  -Kuwait.ti,ab.  -United Arab Emirates.ti,ab.  -UAE.ti,ab.  -Oman.ti,ab.  -Yemen.ti,ab.  -Jordnan.ti,ab.  -Syria.ti,ab.  -Libya.ti,ab.  -Tunisia. ti,ab.  -Algeria. ti,ab.  -Morocco.ti,ab.  - Sudan.ti,ab.  -Somalia.ti,ab.  - Comoros.ti,ab.  -Dijbouti.ti,ab.  -Mauritania.ti,ab. | - help-seeking behavior/  - exp Social Support/  - (help adj3 seek*).ti,ab.  - (help adj3 sought).ti,ab.  - mental health service*.ti,ab.  - Social Support*.ti,ab | -social stigma/  - exp Attitude/  - exp Culture/  - Stigma*.ti,ab.  - attitude*.ti,ab.  - perception*.ti,ab.  - belief*.ti,ab.  - opinion*.ti,ab.  - barrier*.ti,ab  - facilitator*.ti,ab.  - promot*.ti,ab.  - encourag*.ti,ab.  - experienc*.ti,ab. -challeng*.ti,ab.  - motivat*.ti,ab.  - Expectation*.ti,ab.  - drive.ti,ab.  - goal*.ti,ab.  - Intention*.ti,ab.  - Incentive*.ti,ab.  - Purpose*.ti,ab.  Opinion*.ti,ab.  - Outlook.ti,ab.  - perspective*.ti,ab.  - feeling*.ti,ab.  - acceptance.ti,ab.  - taboo*.ti,ab. |

Supplementary file 2: Search strategy
